# Interpretable Classification of Tauopathies with a Convolutional Neural Network Pipeline Using Transfer Learning and Validation against Post-Mortem Clinical Cases of Alzheimer’s Disease and Progressive Supranuclear Palsy

**DOI:** 10.3390/cimb44120406

**Published:** 2022-11-29

**Authors:** Liliana Diaz-Gomez, Andres E. Gutierrez-Rodriguez, Alejandra Martinez-Maldonado, Jose Luna-Muñoz, Jose A. Cantoral-Ceballos, Miguel A. Ontiveros-Torres

**Affiliations:** 1 Tecnologico de Monterrey, School of Engineering and Sciences, Monterrey 64849, Mexico; 2 MAHLE Shared Services, Monterrey 64650, Mexico; 3 Health Sciences Faculty, Universidad Anahuac Mexico Norte, Mexico City 52786, Mexico; 4 National Dementia BioBank, Ciencias Biológicas, Facultad de Estudios Superiores Cuautitlán, Universidad Nacional Autonoma de Mexico, Mexico City 53150, Mexico; 5 Banco Nacional de Cerebros-UNPHU, Universidad Nacional Pedro Henríquez Ureña, Santo Domingo 2796, Dominican Republic

**Keywords:** Convolutional Neural Networks, Guided Grad-CAM, Occlusion Analysis, neurodegenerative diseases, tauopathies

## Abstract

Neurodegenerative diseases, tauopathies, constitute a serious global health problem. The etiology of these diseases is unclear and an increase in their incidence has been projected in the next 30 years. Therefore, the study of the molecular mechanisms that might stop these neurodegenerative processes is very relevant. Classification of neurodegenerative diseases using Machine and Deep Learning algorithms has been widely studied for medical imaging such as Magnetic Resonance Imaging. However, post-mortem immunofluorescence imaging studies of the brains of patients have not yet been used for this purpose. These studies may represent a valuable tool for monitoring aberrant chemical changes or pathological post-translational modifications of the Tau polypeptide. We propose a Convolutional Neural Network pipeline for the classification of Tau pathology of Alzheimer’s disease and Progressive Supranuclear Palsy by analyzing post-mortem immunofluorescence images with different Tau biomarkers performed with models generated with the architecture ResNet-IFT using Transfer Learning. These models’ outputs were interpreted with interpretability algorithms such as Guided Grad-CAM and Occlusion Analysis. To determine the best classifier, four different architectures were tested. We demonstrated that our design was able to classify diseases with an accuracy of 98.41% on average whilst providing an interpretation concerning the proper classification involving different structural patterns in the immunoreactivity of the Tau protein in NFTs present in the brains of patients with Progressive Supranuclear Palsy and Alzheimer’s disease.

## 1. Introduction

Neurodegenerative diseases (NDs), known as tauopathies, constitute a group of more than 20 proteinopathies that represent a major global public health problem; among the most prevalent are Alzheimer’s disease (AD) and Progressive Supranuclear Palsy (PSP). In AD, the Tau protein undergoes modifications that cause its aggregation and the formation of Neurofibrillary Tangles (NFTs), which together with amyloid beta positive plaques are the histopathological hallmark of this disease [1,2,3,4]; likewise, gliosis and neuronal loss are observed [5]. These structures accumulate in the entorhinal cortex and extend to the hippocampus, amygdala, temporal cortex and the isocortex [6]; their accumulation generates alterations in physiological functions that are reflected in the progressive loss of memory and alterations in executive and cognitive functions [7]. Regarding PSP, neuronal loss, gliosis and balloon-shaped and flame-shaped NFT composed of paired helical filaments and straight filaments can be observed, with Tau protein being the main constituent [8]. The histopathological hallmark of PSP is the presence of tufted astrocytes [9] which predominate in cortical and striatal areas [10]. However, NFTs affect the subthalamic nucleus, basal ganglia and brainstem [11]. The clinic of PSP is highly variable, including balance disturbances with falls, rigid hypokinetic syndrome, behavioral and cognitive disorders, ocular motility disorders, secondary disposition, language disorders, dysphasia and sleep disorders [12].

The epidemiological data from [13] highlight the need for an accurate differential diagnosis to establish a prognosis and implement appropriate treatment. The challenge of differential diagnosis is to distinguish the similarities shared by different types of NDs, such as brain atrophy, protein aggregation in specific regions of the brain and protein inclusions detected in the cerebrospinal fluid (CSF) [14]. The efficacy of treatment against neurodegeneration depends on the precise understanding of the molecular mechanisms involved in each disease group, which is not yet fully understood [15].

In the case of tauopathies, pathological protein aggregation is considered a key event. Several research groups [16,17] have concluded that the polymeric behavior of the Tau polypeptide, which constitutes the paired helical filaments that precede the formation of NFT, is due to a series of incorrect post-translational modifications (PTMs) in the Tau protein. These events mainly include phosphorylation, endogenous proteolysis or conformational changes that confer aggregation behavior to the protein in insoluble fibrillar filaments [18,19]. However, these mechanisms continue to be studied in brain tissue in post-mortem cases or by transgenic models of neurodegenerative diseases [20].

Elucidation of the differences between pathological PTMs in the Tau protein that leads to its fibrillar polymeric form is fundamental to understanding the pathogenesis and differential diagnosis between the different tauopathies, representing a critical challenge for therapeutics [21].

Machine and Deep Learning, specifically Convolutional Neural Networks (CNNs), have been used to address the problem of differentiating NDs in medical imaging, such as Magnetic Resonance Imaging (MRI), Computerized Tomography (CT) and Positron Emission Tomography (PET), which are noninvasive means for detecting changes in brain function [22,23,24]. These imaging modalities provide a macroscopic view of brain atrophy. Alternatively, the way to explore at the molecular scale is based on immunofluorescence post-mortem brain (IPMB) microscopy, which is a technique that uses antibodies directed against chemical events occurring in specific proteins to visualize them in the cells of the tissues studied [25]; thus, the analysis of NDs’ pathogenesis depends on these techniques and experimental protocols to provide us with a molecular understanding.

The ability of immunofluorescence to discriminate between cells, organelles or molecules within tissues and to analyze their interactions through the obtained images makes it an ideal data format for more advanced computational analysis [26]. In particular, the use of Deep Learning (DL) methods for the classification and differentiation of tauopathies may lead to finding particular features of the behavior of the Tau protein in the formation of NFTs, which currently only depends on the visual appreciation of biochemical and biomedical experts with a possible risk of subjectivity among the different criteria for interpretation.

Deep Learning is a computational paradigm that has been exploited for medical image classification [14,22,27,28], specifically CNNs have contributed significantly to the areas of medical image understanding and many CNN-based approaches lead the way in many image understanding challenges for diseases such as cancer, autoimmune diseases, stroke lesions and brain diseases [28]. Moreover, the use of Explainable Artificial Intelligence (XAI) algorithms, while scarce, has provided a way to elucidate the behavior of deep neural networks [29]. Moreover, DL models have outperformed human experts in many image understanding tasks, e.g., CNN-based models such as CheXNet for classification of ailments of the chest have achieved better results compared to the average performance of human experts [30,31].

Within the context of DL, Transfer Learning is a technique that has also been exploited for medical image classification [32]. It consists of taking a pre-trained neural network, on a source domain, such as the dataset ImageNet [33], which contains more than fourteen million labeled images with more than twenty thousand categories [34], and taking that pre-trained model to a different domain, usually with a limited number of images. For example, in neurosciences, Transfer Learning based on AlexNet [35], was used to detect Alzheimer’s disease using the dataset ImageNet in [36]. Additionally, Zhuang et al. [33] show that most classification problems on medical images use some variation of Transfer Learning with fine-tuning.

### 1.1. Immunodetection and Fluorescence Miscoscopy

The field of DL for classification of immunofluorescence microscopy imaging has been widely studied for HEp-2 cell classification. Rahman et al. [37] provide an extensive review of DL models developed for classification of HEp-2 cells between the years 2013 and 2019. Architectures such as ResNet-50 without a pre-processing step have achieved an accuracy of 98.42% [38]; LeNet-5, AlexNet and GoogleNet along with contrast stretching and histogram equalization pre-processing techniques have achieved an accuracy of 98.17% [39].

Neurons have been classified in immunofluorescence images of rat brains, where CNN showed better performance than Principal Component Analysis (PCA) with a Support Vector Machine. However, this research explains that their model may not be suitable for the hippocampus region given its dense neuronal population [40]. ResNet-101 architecture has been used to classify immunofluorescence images of kidney biopsies with an accuracy of 79% [41]. Myelin detection for classification of immunofluorescence images has also been performed, testing 23 Machine Learning (ML) algorithms with the highest accuracy encountered for Custom CNN and Boosted Trees methods with 98.84% and 98.46%, respectively [42]. However, although different studies focus on immunofluorescence images, none of them address the study of tauopathies from IPMB images.

The only related work found on classification of immunofluorescence post-mortem brain imaging is presented by Alegro et al. [43], who propose a method for automated cell counting based on segmentation followed by classification of cells using dictionary learning and sparse coding. The authors explain that they did not use DL models because they needed to train with small sample sets. The accuracy of the classification was expressed in terms of recall and precision, which are 71% and 25%, respectively. However, despite being performed on the same image domain, this research is not comparable to ours because its main objective was segmenting and counting, not classifying.

### 1.2. Neurodegenerative Disease Classification Using Machine and Deep Learning

Lin et al. [44] classified different spectrums of neurodegenerative diseases using plasma biomarker levels. The authors perform dimensionality reduction and then test seven different ML models, with Random Forest being the best model with an accuracy of 86%. Tang et al. [29] classified amyloid-beta pathologies by immunohistochemistry in human brain tissue. The authors use a customized CNN for the classification of three types of beta-Amyloid plaques. This paper also performs an interpretability study of the DL model using Guided Grad-CAM activation mapping and feature occlusion studies. This research obtained an overall accuracy of 97.3% based on this polypeptide, which, like Tau, are considered the main proteins involved in the pathogenesis of Alzheimer’s disease.

Gao et al. [45] provide a DL method for classification of CT images into classes: Alzheimer’s disease, lesion and normal aging. The architecture used is both a 2D and 3D CNN and it yields an average accuracy of 87.6%. Alternatively, Rohini et al. [14] propose a model of classification of Alzheimer’s disease, mild cognitive impairment (MCI), Pre-MCI and healthy controls based on neuron degeneration. The authors assemble a Machine Learning model with SVM, K-nearest neighbors and Gaussian Naive Bayes classifier. The yielded accuracy was 88.5%, whereas the features used for training the model were thickness and volume of the brain on the images. Singh et al. [27] also used MRI images for classification of Parkinson’s disease versus scans without evidence of dopaminergic deficit and healthy controls. The authors use an SVM model that implies an accuracy of nearly 100%; however, the dataset tested comprised only 150 images; therefore, it is unsure if the method works on larger datasets.

As the conclusion of the literary review, IPMB images have not been used for studies of classification of NDs. While MRI and CT scan at a generalized level and are based on morphological data of brain tissue, IPMB images are based on brain tissue but at a molecular level, which is key to understanding the pathogenesis of NDs. Therefore, the design of a classification model among the different tauopathies, with a focus on aberrant PTMs suffered by the Tau polypeptide, constitutes the challenge of the present investigation.

In this study, we modeled the different biomarkers concerning pathological PTMs in Tau polypeptide in the hippocampal and entorhinal cortex regions of the brain using a DL and Transfer Learning pipeline that classifies AD and PSP tauopathies on IPMB images, provided by the National Biobank of Dementias of the National Autonomous University of Mexico (UNAM). From a broad range of DL architectures, we developed the ResNet-IFT architecture, which is a ResNet-50-based architecture that proves to be efficient for obtaining models for classifying IPMB images. The models developed in this study test whether Transfer Learning or Transfer Learning and fine-tuning are helpful tools to develop the pipeline. This pipeline is followed by Guided Grad-CAM and Occlusion Analysis algorithms in order to obtain the actual differences in Tau polypeptide that lead to the classification of each disease. To our knowledge, the present work is the first one proposed to classify NDs from parameters computed by IPMB images.

## 2. Materials and Methods

The following section presents the specifications of the IPMB images used for the project. We also introduce the datasets we constructed to carry out the experimentation. Next, we present four distinct architectures and a comprehensive comparison of their performances to obtain the best classifier. Within the section, we provide a brief explanation concerning the ResNet models and Transfer Learning for DL. Finally, we present the specifications and results from implementation of XAI algorithms, Guided Grad-CAM and Occlusion Analysis, to interpret the most significant regions of the IPMB images for an accurate classification of AD or PSP.

### 2.1. IPMB Images

The IPMB images used for the research were obtained in a collaborative project between the National Dementia Biobank of the UNAM and the Bioengineering Department of the Tecnologico de Monterrey. The images were obtained entirely from post-mortem tissues of patients with AD and PSP. All data were obtained following current laws, regulations and guidelines, such as sharing anonymized data that does not contain information that would establish the identity of individual deceased subjects.

Delving deeper into the specifications of the brains used to obtain the IPMB images:

- Four different brains were used.
- The brain areas used were Hippocampus CA1 and Entorhinal cortex.
- Two brains with diagnosed AD were used—one of a 90 year old female and another of an 81 year old male.
- Two male brains with diagnosed PSP were used—one of a 75 year old and another one of a 85 year old.
- The tissues of patients with AD used were of the Braak 5–6 stages.

The IPMB images are a visual representation of the interaction of fluorochrome-coupled antibodies with their epitopes on the specific protein chemical structure. The images also represent molecules with chemical interactions of fibrillar forms such as NFT in the brain of patients. Experiments with three different biomarkers were used, resulting in four-channel imaging:

- Green channel: Inmunodetection with AT8 mouse IgG antibody (MN1020, Invitrogene) against Tau protein. AT8 antibody detects phosphorylations of Tau protein in amino acids: Serine 202 and Threonine 305. The presence of phosphates translates into chemical changes that give protein Tau aberrant behaviour.
- Red channel: Inmunodetection with Thiazine red, which is a molecule that binds to fibrillar insoluble structures of protein polymers. This molecule specifically binds to protein Tau in its polymer conformation.
- Blue channel: Inmunodetection with pS396 rabbit IgG antibody against Tau protein [46]. The 396 antibody locates a phosphorylation in Serine 396 amino acid, which is known as a chemical change in protein Tau that associates with the formation of NFT.
- Merge channel: Visualization of the green, red and blue channel images together into one image.

Images were obtained using a 100× oil-immersion plan Apochromat objective (NA 1.4). Ten to fifteen consecutive single sections were sequentially scanned at 0.8–1.0 μm intervals for two or three channels throughout the z-axis of the sample.

It is important to note that our images are captured with the same criteria and we block the nonspecific background signal when incubating the corresponding antibodies in the immunofluorescence. Moreover, it is important to highlight that the obtention of our dataset has been a work that has taken over ten years.

For the development of the project, a pre-processing stage was not needed, the images were processed as they were delivered by the experts, already labeled.

### 2.2. Datasets

**Figure 1:**
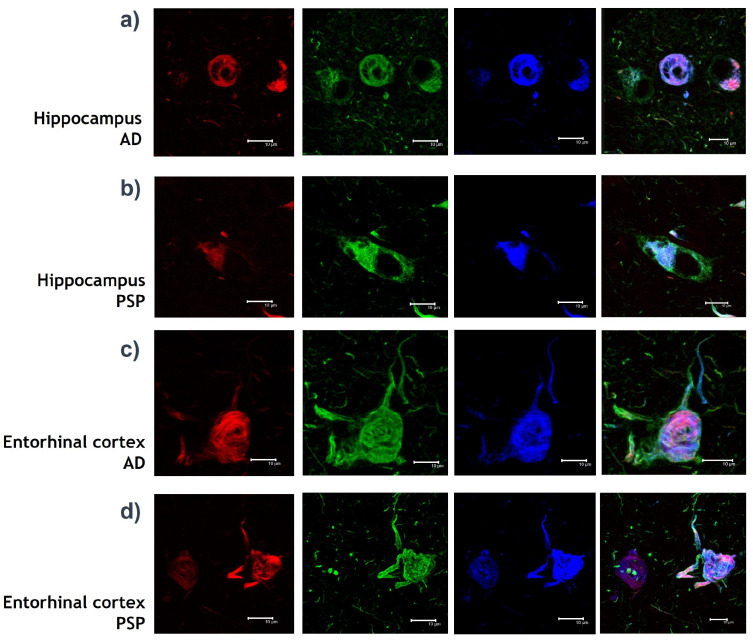
Representative images organized by class and by dataset. (**a**) Images from D1 and D3 corresponding to Alzheimer’s disease (AD). (**b**) Images from D1 and D3 corresponding to Progressive Supranuclear Palsy (PSP). (**c**) Images from D2 and D3 corresponding to AD. (**d**) Images from D2 and D3 corresponding to PSP. From left to right, we can see the IPMB images with the respective green, blue, red and merge channels for different Tau polypeptide biomarkers in both tauopathies.

Three datasets were formed to evaluate the performance through experimentation, as shown in Table 1. The class balance maintained a ratio of 54–46%, on average. The main purpose of the image distribution among the datasets was to obtain insights of pathologies of PSP and AD according to the brain area: hippocampus or entorhinal cortex. At the same time, we had the purpose of evaluating the classification models and their ability to generate determinations regardless of having a division per brain area. In Figure 1, we can observe a random sample of images according to the datasets in Table 1.

### 2.3. Model Development and Training

In order to explain our main contribution, i.e. the classifier and the model’s interpretability merit from XAI algorithms, we briefly address first the theory behind our choices of architectures to test.

It is commonly understood that DL models work best when copious amounts of annotated data are available; however, for our research, even our largest dataset (D3) is relatively small (over 1300 images). Therefore, in order to achieve optimal performance, we considered a wide range of CNN architectures, starting with a multilayer perceptron and then increasing the model complexity, up to a pre-trained ResNet50 architecture using Transfer Learning and fine-tuning. In the related work, previously presented, artificial neural networks and Deep Learning models have been used for the classification of immunofluorescence images and the classification of neurodegenerative diseases. Therefore, we decided to test two artificial neural networks with different depths and two Deep Learning architectures with different complexity.

Given the limitations of our datasets, we decided to use Transfer Learning, thus initializing the weights from an ImageNet pre-trained model, rather than randomly, since this would help extracting features from the IPMB images dataset. Moreover, Transfer Learning has been proven to save time and achieve a better performance than training an entire model from scratch [47]. Moreover, Transfer Learning improves the learning skills in the target task given the knowledge from the source task [48].

#### 2.3.1. ResNet Models

Since the introduction of ResNet models by He et al. [49], ResNet-based architectures have shown good convergence behaviors [50], particularly in medical imaging classification [51] as well as in immunofluorescence imaging [38,39], where these models have obtained high accuracies. ResNet models are based on the idea of having convolution blocks and identity blocks joined by shortcuts in order to avoid the vanishing gradient problem [49]. Each convolution block and identity block is composed by repetitions of convolution, batch normalization and activation. ResNet-50 contains convolution and identity blocks that together form 49 convolutional layers with a fully connected layer at the end of the network. ResNet-50 was chosen among the broad options of pre-trained architectures because, given their residual mapping and shortcut connections, it consistently leads to better results compared to very deep plain networks both in accuracies and in training times [49].

#### 2.3.2. Transfer Learning

As we briefly explained in the Introduction, Transfer Learning uses a model trained on a source domain for a specified task and then re-uses that model for a different task [52]. It is important to highlight that there are different Transfer Learning categories:

- Homogeneous Transfer Learning: Source and target feature spaces are the same.
- Heterogeneous Transfer Learning: Source and target feature spaces are different.

For the development of this project, we define two levels of Transfer Learning:

- Transfer Learning: Initializing the weights of the model using a pre-trained architecture on the ImageNet dataset. Then, we preload these weights to train the entire architecture with our developed dataset.
- Transfer Learning and fine-tuning: Initializing the weights of the model using a pre-trained architecture on ImageNet dataset, but training only the last convolution block and the final fully connected layer with our developed dataset, for some epochs. Afterwards, we unfreeze the entire architecture and continue to train the model for additional epochs.

From the explanations above, it should be noted that we follow a heterogeneous Transfer Learning approach, since we will use ImageNet pre-trained architectures for the classification of IPMB images. In addition, we tested both Transfer Learning and Transfer Learning plus fine-tuning.

#### 2.3.3. Classifier Development and Testing

To accomplish the goal of developing the most suitable model for IPMB images classification, we developed four different DL architectures:

- Sequential CNN: A Multi-Layer Perceptron with three fully connected linear layers with 18, 8 and 2 neurons. L2 regularization was used for the last linear layer. The training was performed for 30 epochs.
- Simple CNN: A CNN with 3 convolution layers, starting with 16 filters, then 32 and lastly 64 filters of 3 × 3 kernels. Each convolution was followed up by a max pooling operation with a 2 × 2 window. Then, we reduced the images to a one-dimensional vector and used two fully connected layers with 200 and 2 neurons, respectively. We applied a dropout layer with a rate of 0.5 between these two fully connected layers. Each convolution and linear operation was performed with L2 regularization. The model was trained for 30 epochs.
- ResNet-IFTF: The architecture ResNet-50, as provided in Keras, was used. This architecture is pre-trained on the ImageNet dataset. Transfer Learning and fine-tuning were applied for the development of this model. We froze the entire model up to the last activation layer encountered and added a convolution layer with 512 filters with kernels of size 1 × 1, a batch normalization layer, an activation layer, a global average pooling layer and an output layer with 2 neurons and L2 regularization. The model was trained for 15 epochs with the frozen part of the architecture and then for 10 additional epochs unfreezing the entire model.
- ResNet-IFT: The architecture ResNet-50 from Keras was used. This architecture was pre-trained on the ImageNet dataset. For this model, we used Transfer Learning using the entire pre-trained RESNET without any further fine-tuning. The entire architecture was trained for 15 epochs. We added a global average pooling layer and an output layer with L2 regularization. In comparison to the original ResNet-50 architecture, we eliminated the flattening layer between the global average pooling layer and the dense layer.

**Figure 2:**
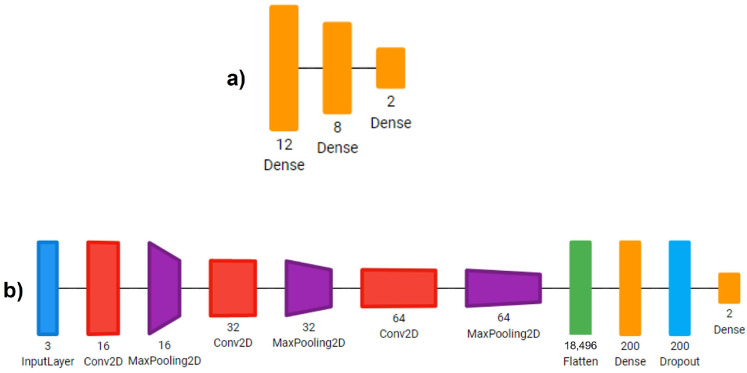
Simplest architecture designs used in this study. (**a**) Sequential CNN architecture. (**b**) Simple CNN architecture (Diagrams of the architectures were developed using Net2Vis [53] tool for visualizing Deep Learning models).

**Figure 3:**
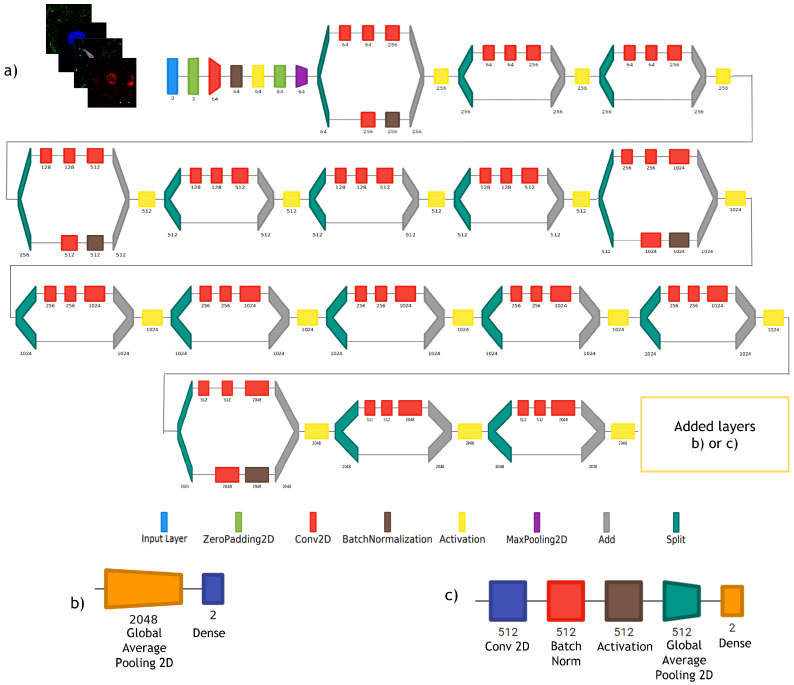
ResNet-50-based architecture designs used in this study. (**a**) Base model of ResNet-50 from Keras until the last activation layer of the last convolution. (**b**) Layers added at the end of the base model for development of ResNet-IFT. (**c**) Layers added at the end of the base model for development of ResNet-IFTF. (Diagrams of the architectures were developed using Net2Vis tool for visualizing Deep Learning models).

All neural network models were developed and trained in the open source package Keras of Tensorflow. The threshold used for classification in all neural networks was 0.5. In Table 2 we can see the summary of the specifications of the layers of each of the four architectures that we previously described. In Figure 2 and Figure 3, we present a visual representation of the architectures. As we can see in Figure 3, for the ResNet-IFTF architecture, we were required to add an additional convolutional block (convolutional layer + batch normalization layer + activation layer) in comparison to the original ResNet-IFT architecture. The additional convolutional block was required to perform the interpretability study, in terms of coding.

### 2.4. Interpretation by XAI Algorithms

The XAI algorithms chosen to aid in the interpretation of our models are Guided Grad-CAM and Occlusion Analysis, which allow us to test both a back-propagation-based method and a perturbation-based method [27].

#### 2.4.1. Guided Grad-CAM

**Figure 4:**
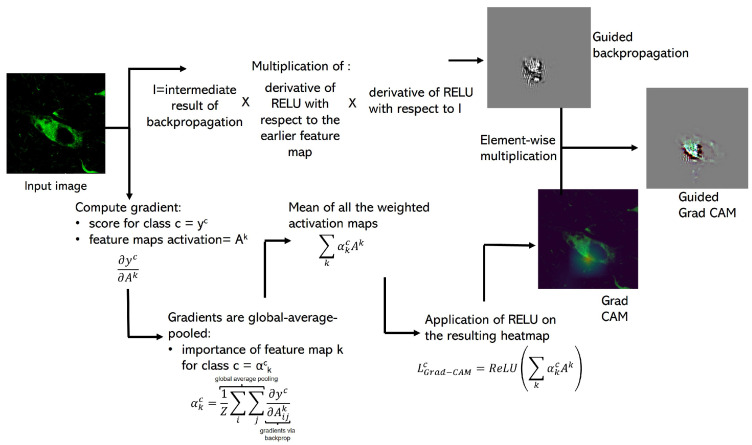
Guided Grad-CAM study diagram. This process has three stages. Stage one: Grad-CAM computation. The input image enters the classifier previously trained and it is forward-propagated until the last convolution layer. Here, we computed the gradients of the scoring class for the activations of the feature maps of the last convolution layer. Then, the gradients flowing back are global-average-pooled to obtain a weight of each feature map for a target class. Each activation map is multiplied by its corresponding weight and we obtain the average. Finally, we apply ReLU on the resulting visualization to keep only positive influence on the output map. Stage two: guided back-propagation computation. The input image enters the classifier previously trained and it is forward-propagated until the last convolution layer. A current intermediate result of back-propagation is multiplied by the derivative of ReLU with respect to the earlier feature map, which is also multiplied by the derivative of ReLU with respect to the current intermediate result of back-propagation. Stage three: Guided Grad-CAM computation. The output of Grad-CAM and guided back-propagation are element-wise multiplied and we obtain the Guided Grad-CAM visualization.

In order to provide additional interpretations of the results, we look into the CNN’s internal logic using the Guided Grad-CAM algorithm [54]. This visualization technique is a combination of Grad-CAM and guided back-propagation, obtaining as a result a technique that is class-discriminative, localizes relevant image regions and highlights fine-grained pixels that contribute to the classification of an image. Guided Grad-CAM uses the gradient information flowing into the last convolution layer of the CNN with the aim of understanding the importance of each neuron for a decision of interest. In Figure 4, we provide an explanatory diagram of this technique. We employed an open-source implementation of Guided Grad-CAM by Khandelwal [55].

#### 2.4.2. Occlusion Analysis

**Figure 5:**
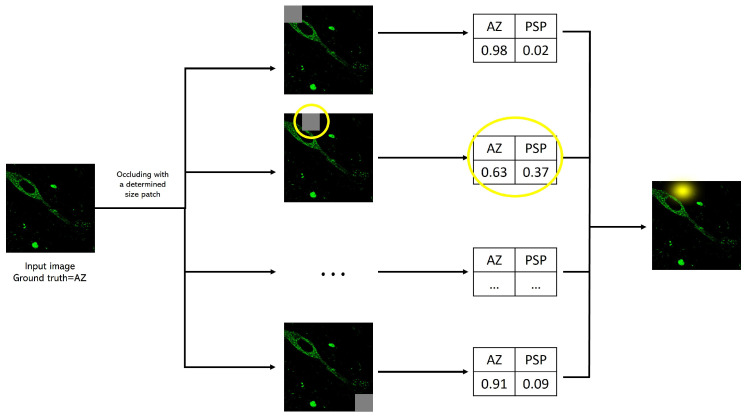
Occlusion sensitivity study diagram. The input image enters the previously trained classifier; then, a patch is placed covering a certain part of the figure and a prediction score is made. The process of placing a patch in another region of the image and generating a score continues until the patch has been placed throughout the image. Regions where the prediction score changed considerably are highlighted with a yellow gleam.

This technique passes a fixed-size patch across the image, evaluating the class prediction for each patch location in the picture [56]. Figure 5 presents an explanatory diagram of this process. The purpose of this process is to determine image areas that, when covered by a patch, considerably affect the predicted class. For this study, we used the Python library tf-explain [57] and defined a squared patch of 20 pixels.

### 2.5. Evaluation Metrics

For the evaluation of the classification models, we used the metric accuracy (number of correct predictions/total predictions) as our guide to define a successful model. This metric was chosen because our datasets are balanced. We used 10-fold cross validation to determine the standard deviation of the models developed per architecture. Each dataset was split into 80% for training and validation and 20% for evaluation or testing.

The Guided Grad-CAM and the Occlusion experiments were evaluated by confirmation by human experts. For the XAI experiments, we were interested in obtaining an interpretation and insights on the importance of a certain prediction.

## 3. Results

The following section describes the accuracy metrics obtained for the models developed with the four CNN architectures previously described. Moreover, this section stresses the best classification model comparing the probability scores for a determined class. Finally, we present the findings for the interpretability study.

### 3.1. Classification Models per Dataset

**Figure 6:**
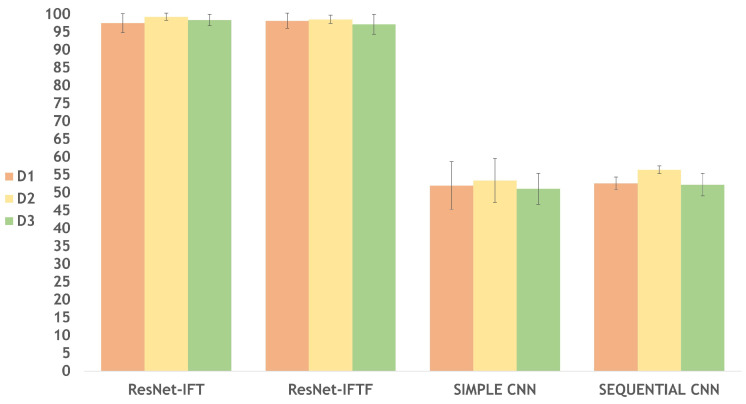
Accuracy performance per architecture. The ResNet-IFT and ResNet-IFTF architectures reach an accuracy between 97.2% and 99.29%; however, ResNet-IFT gives the best performance for 2 of the 3 datasets developed for this study.

As explained in the previous section, we developed four architectures and tested their accuracy to obtain the most suitable model for IPMB image classification. For each dataset (D1, D2 and D3), we tested each of the four architectures, thus developing a total of 12 models. In Table 3 and Figure 6, we can see that the architecture ResNet-IFT obtains the highest accuracy for the models developed using D2 and D3; however, ResNet-IFTF obtains the best accuracy for the model developed with D1. The Sequential and Simple architectures do not reach an accuracy greater than 56.41% and 53.42%, respectively. We can also observe the largest standard deviation from the Simple architecture, whilst the smallest one is obtained from the ResNet-IFT.

**Figure 7:**
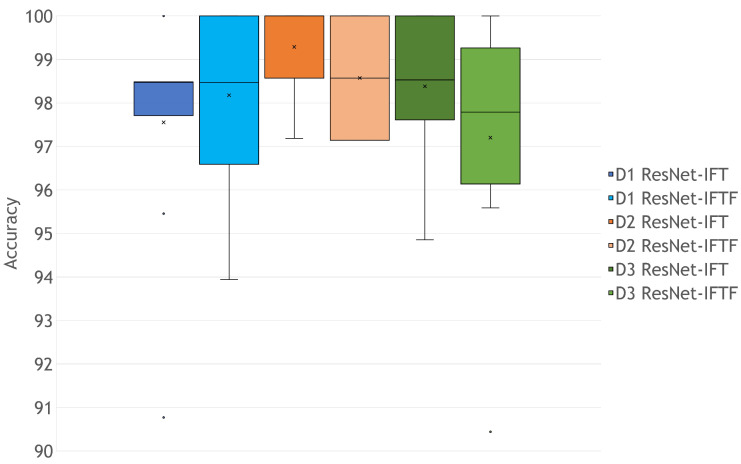
Accuracy analysis for ResNet-IFT and ResNet-IFTF. The quartile calculation used exclusive median; the x symbol shows the mean values.

Since the accuracies obtained for the models using Sequential and Simple architectures are the lowest, but the accuracy of the models using ResNet-IFT and ResNet-IFTF are similar, we were able to visualize the effect of applying a level of Transfer Learning on a classification task for IPMB images. We decided to explore further the statistical meaning of the results of the models developed with the ResNet architectures. As we can see in Figure 6, the models with ResNet-IFT and ResNet-IFTF have similar performance; however, in Figure 7, we can see that with ResNet-IFTF, the models have dispersed results in 10-fold cross validation, whereas with ResNet-IFT (even for D1) smaller data dispersion is achieved. Moreover, the accuracy of the model with D1 in ResNet-IFT is affected by the presence of outliers; nonetheless, its accuracy median is 98.47%.

### 3.2. Rigor of the Classification

**Figure 8:**
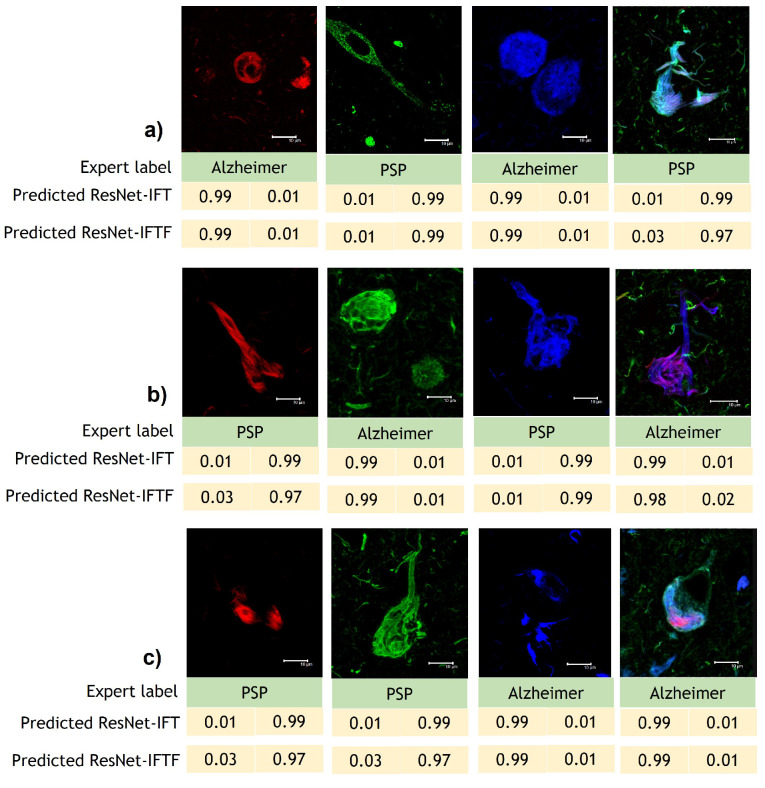
Sample of images for prediction analysis with ResNet-IFT and ResNet-IFTF. (**a**) Models developed using D1. (**b**) Models developed using D2. (**c**) Models developed using D3.

As a final experiment to determine the best performing model between ResNet-IFT and ResNet-IFTF, we tested a random sample of images in order to obtain the prediction value per class, as shown in Figure 8; while with ResNet-IFT, we achieve prediction scores above 99% for each image, with ResNet-IFTF the model results fluctuate between 97 and 99%. Therefore, we selected the models obtained with ResNet-IFT to carry out interpretability analyses.

**Figure 9:**
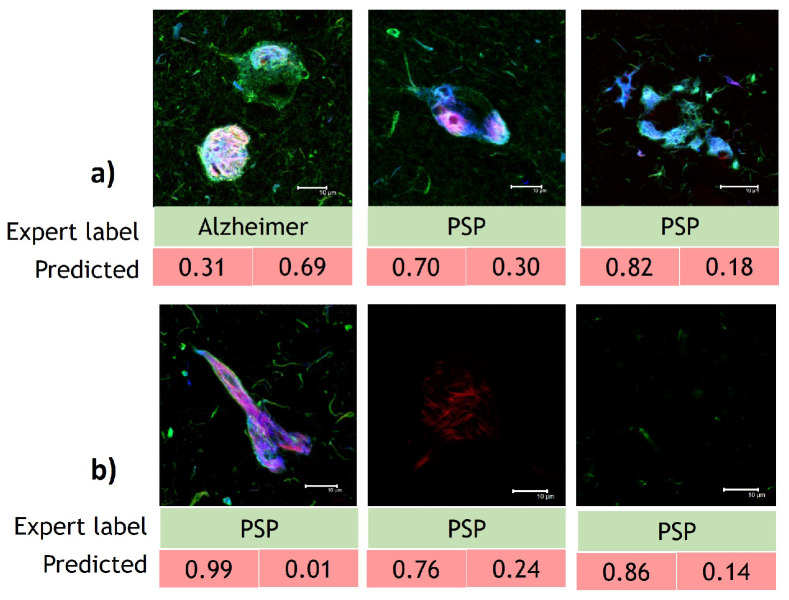
Unique misclassified images using ResNet-IFT architecture. (**a**) Model developed using D1. (**b**) Model developed using D2.

Furthermore, in Figure 9, we can see that the ResNet-IFT architecture misclassified only three images from D1 and D2, with only three D1 misclassified fusion channel images. In D2, ResNet-IFT incorrectly classified one green channel image, one red channel image and one fusion channel image. It is important to note that ResNet-IFT trained on D3 did not misclassify any images.

### 3.3. Interpretability Study

The DL pipeline obtained from ResNet-IFT was saved and then loaded for the Guided Grad-CAM and Occlusion Analysis algorithms to obtain the final visualizations.

**Figure 10:**
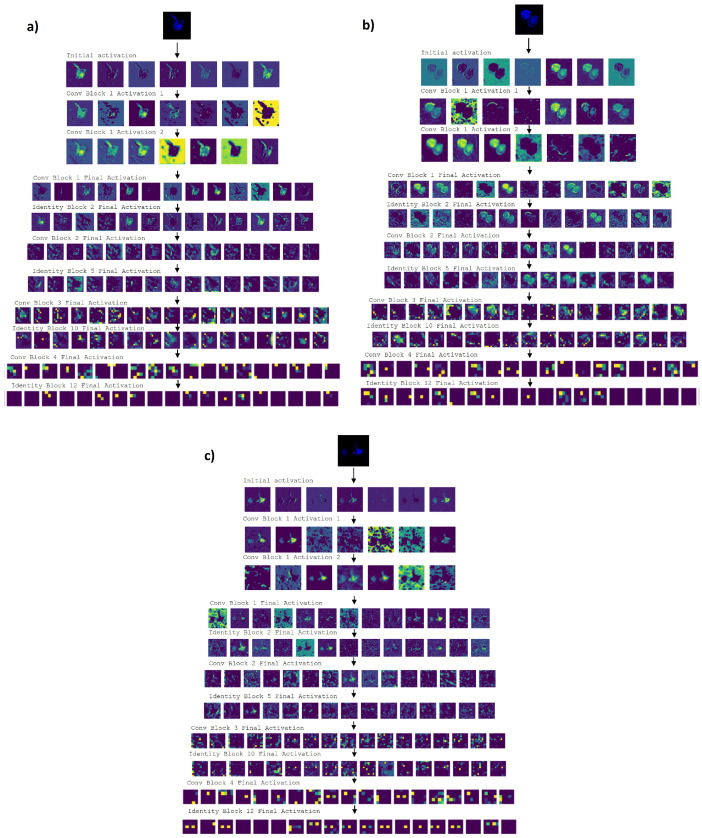
Activations throughout the model ResNet-IFT testing. (**a**) Activations of a PSP image using ResNet-IFT with D2. (**b**) Activations of an AD image using ResNet-IFT with D1. (**c**) Activations of an AD image using ResNet-IFT with D3. From the fifth row of (**a**–**c**) we can see that features that are not visible in the original image start to be taken into account for the activation of the models.

Firstly, we obtained the visualizations of the activations of the entire models of ResNet-IFT trained on D1, D2 and D3. As we can see in Figure 10, the earlier layers of each model are mainly activated by either the colored part of the image or the entire background of the image. As we go deeper in the model, we can see that, even though the colored portion of the images is always a significant factor to be classified as AD or PSP, there are also portions of the background that are activated. However, it is interesting to note that some portions of the activated background show non-immunoreactive zones that are not even colored in the original image. These activations were obtained using the Keract [58] open source library.

We are able to see spots of non-immunoreactivity that are not colored in the original image thanks to the filters learned by the models. The convolution operation in CNN is an element-wise multiplication followed by a sum, between an input datum and a filter that gives us an output feature map. The convolutional layers execute the convolution operation with the filters learned by the models, in order to perform feature extraction. As the depth of the CNN increases, the complexity of the features learned by the CNN increases. Therefore, even though we are not able to appreciate completely some spots of non-immunoreactivity in the original image, these are reflected as a set of broad features that the models are able to abstract from the original image.

From our experimentation, we could identify features of Tau protein that differentially associate between the hippocampal region and the entorhinal cortex of the brain. Although the results coincide with the studied areas of tangles by neurophysiologists, it is noteworthy that our CNN pipeline also located other discriminative criteria outside the zones of elongation of the polymeric filaments of the Tau protein or outside the body of the neurofibrillary tangle.

**Figure 11:**
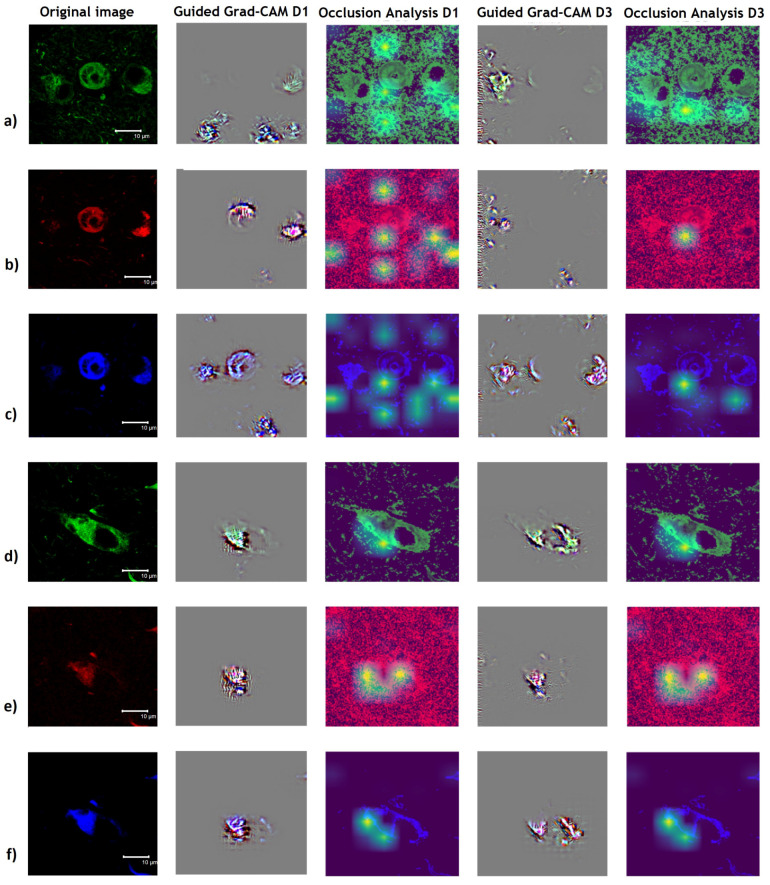
Model interpretability studies using XAI techniques for PSP and AD classification in the hippocampus region of patients with tauopathies. (**a**) Guided Grad-CAM analysis (second and fourth column) for AD classification of green channel highlights NFT in the southern (for D1) and western (for D3) periphery of the image. Occlusion Analysis (third and fifth column) highlight the southern and northern periphery (for D1) and only the southern periphery (for D3) of the image. (**b**) Guided Grad-CAM analysis (second and fourth column) for AD classification of red channel highlights neurofibrillary tangle at the center (for D1) and periphery (for D3) of the image. Occlusion Analysis (third and fifth column) highlights the southern and northern periphery as well as the center (for D1) and only the center of the image (for D3). (**c**) Guided Grad-CAM analysis (second and fourth column) for AD classification of blue channel highlights neurofibrillary tangle at the center and southern periphery (for D1) and periphery (for D3). Occlusion Analysis (third and fifth column) highlight the southern and northern periphery as well as the center (for D1) and only the center and southern periphery (for D3). (**d**–**f**) Guided Grad-CAM analysis (second and fourth column) for PSP classification highlights mainly over all the originally colored middle portion of the image (for D1 and D3). Occlusion Analysis (third and fifth column) highlights the center of the image (for D1 and D3). In the green channel (**a**,**d**) immunoreactivity of the AT8 antibody directed against the biomarker for dual phosphorylation at serine 202 and threonine 305 of the Tau polypeptide is observed (**a**,**d**). The red channel (**b**,**e**) shows staining with Thiazine red dye for fibrillar forms of pathological Tau aggregates. The blue channel (**c**,**f**) shows immunoreactivity of the pS396 antibody directed against the biomarker for phosphorylation at amino acid serine 396 of Tau protein.

In Figure 11, we can see that the features highlighted for Guided Grad-CAM are consistent with immunoreactivity in NFT structures. For example, for AD prediction (Figure 11a–c), we can see that the colored pixels are crucial for the prediction. However, it is interesting to note that, although the same quadrant structure presents immunoreactivity with AT8 antibody, 396 antibody or Thiazine red, the fine-grained details that Guided Grad-CAM highlights are different from those highlighted by the immunoreactivity. For the green channel, the stained tangles in the periphery seem to be significant, whereas for the red channel the tangle with circular morphology in the center seems to be decisive for classifying the image as AD.

Regarding the blue channel, which correspond to pS396, it shows a relationship between the immunoreactive tangles in the periphery and the one located in the medial zone with round morphology. However, the criteria obtained with the model developed for D3 are not as enriching because they seem to point more to the immunoreactive structures in the periphery rather than to the central tangle. We find this result very interesting because of the implications of the phosphorylation of serine 396 of the Tau protein as an event considered closely associated in the final stages of NFT formation.

From Figure 11a–c, i.e., the Occlusion Analysis study, we can confirm that the model trained only on D1 images locates more significant regions that contribute to the classification of tauopathies. However, the Occlusion Analyses for D1 and D3 coincide with the Guided Grad-CAM in establishing that the pS396 biomarker in the blue channel is associated to the periphery and center of the image with significant criteria, the red channel for the insoluble fibrillar forms has less peripheral presence of the image and the green channel associates less towards the periphery. Moreover, we can see that the model trained on D1 is less selective for the relevant structures of the image because in D3 we also have images of the entorhinal cortex and not just the hippocampus.

For PSP classification in the hippocampus, we can observe that there is a correlation in the relevant part of the image that does not fully agree with the immunoreactivity detected with the corresponding antibody (Figure 11d–f) because there are areas other than immunoreactivity that ResNet-IFT considers relevant and that may represent a differentiating factor from the point of view of pathogenesis. It is important to highlight that for both models developed for D1 and D3, the Occlusion Analysis and the Guided Grad-CAM visualizations give similar results.

**Figure 12:**
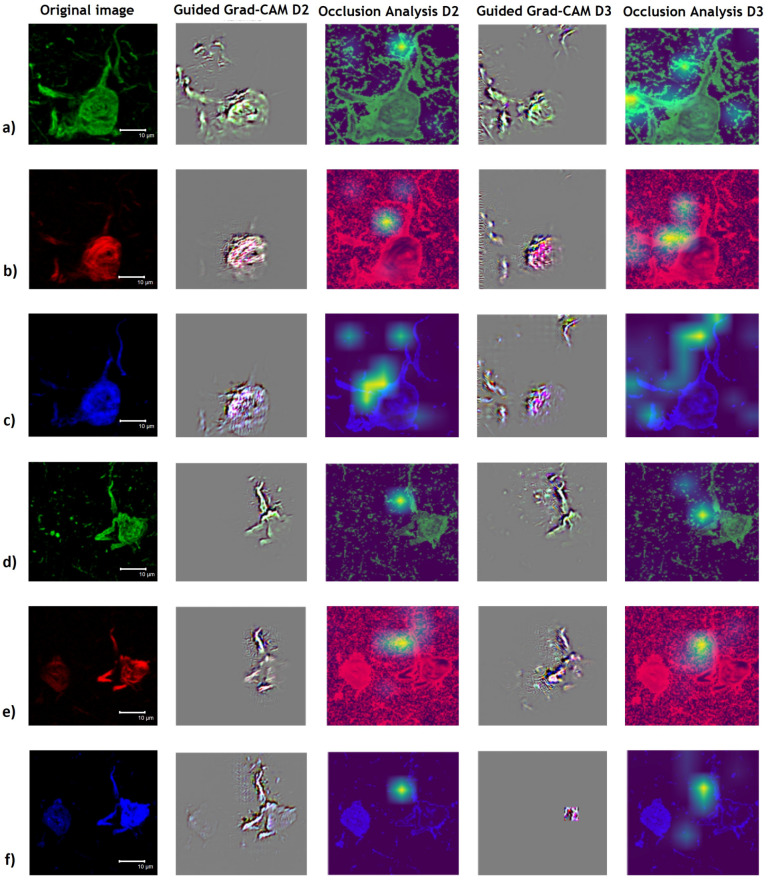
Model interpretability studies using XAI techniques for PSP and AD classification in the entorhinal cortex region of patients with tauopathies. (**a**–**c**) Guided Grad-CAM analysis (second and fourth column) for AD classification highlights the most prominent NFT of the image; however, for D2 mostly the entire NFT is spotted and for D3 the southwest region of the tangle is spotted. The Occlusion Analysis (third and fifth column) highlights the northern and center region (for D2 and D3) of the image. (**d**–**f**) Guided Grad-CAM analysis (second and fourth column) for PSP classification mainly highlights all the originally most prominent immunoreactive middle portion of the image (for D2 and D3). Occlusion Analysis (third and fifth column) highlights the center of the image near the apex of the central NFT (for D2 and D3). In the green channel (**a**,**d**), immunoreactivity of the AT8 antibody directed against the biomarker for dual phosphorylation at serine 202 and threonine 305 of the Tau polypeptide is observed (**a**,**d**). The red channel (**b**,**e**) shows staining with Thiazine red dye for fibrillar forms of pathological Tau aggregates. The blue channel (**c**,**f**) shows immunoreactivity of the pS396 antibody directed against the biomarker for phosphorylation at amino acid serine 396 of Tau protein.

In Figure 12, we can see that for the images of the entorhinal cortex, the most prominent visualized tangle always contributes to the prediction of either AD or PSP. However, other areas of fibrillar growth similar to the neuropil are always relevant to the biomarker in the green channel, as we can see in Figure 12a,d (second and fourth column). The results of the Occlusion Analysis are very similar despite the dataset used to train the model. These results highlight structures not localized to the NFT growth body.

## 4. Discussion

From our comprehensive experimentation, we can see that the Transfer Learning model displays the best prediction performance. Thus, this is the model we used to implement interpretability models to analyze and identify AD and PSP tauopathies from IPMB images. In addition, Guided Grad-CAM and Occlusion Analysis help us to obtain information about the molecular pathogenesis of the tauopathies that was not recognized in a conventional interpretation.

### 4.1. Transfer Learning Model versus Fine-Tuning Model

From our initial experimentation, we can see that the Sequential and Simple CNN architectures do not generalize properly or abstract enough information to learn the features of the IPMB images dataset. We decided to start with a three-layer MLP since we were dealing with medical images from which we did not know the complexity for classification. As we can see, the spatial information is lost in this model and therefore it is not a good fit for the IPMB images. However, it is interesting that the Simple CNN-based models behaved slightly worse, as shown in our standard deviation analyses we carried out. However, in this case the Simple CNN-based models were affected by the random weight initialization instead of using pre-trained weights.

In the case of the models developed using Transfer Learning and Transfer Learning plus fine-tuning, the Transfer Learning model using pre-trained weights of the ImageNet dataset achieved the best results. According to Guo et al. [59], it is not clear if fine-tuning to the last contiguous layer is de facto the best option in all applications. The reason is that ResNets can be considered not as a large deep network, but rather as sets of shallow networks [60]. Therefore, freezing a part of the architecture means that the ensemble effect diminishes the assumption that early or middle layers should be shared with common low-level or mid-level features. Moreover, Pan et al. [47] explain that the phenomenon of “negative transfer” occurs when the source domain of the model, in this case the ImageNet dataset, does not match the target domain, in this case the IPMB images. Moreover, Peng et al. [61] obtain as insight that fine-tuning with a smaller dataset gives a better result than with a larger dataset. The authors also explain that with a larger dataset training the entire model has a better output than fine-tuning. This can give us an insight into the minimum amount of images for the Transfer Learning without fine-tuning to be more effective than fine-tuning it.

As we can see in Figure 7, the models for the hippocampus obtained the most significant accuracy median with fine-tuning; however, with only Transfer Learning, it had the presence of some outliers. Moreover, for the entorhinal cortex, the improvement using only Transfer Learning is noticeable; therefore, the minimum number of images required to favor our Transfer Learning model oscillates between 656 and 702 images, approximately. This is supported taking into account that for D1 (656 images) Transfer Learning with fine-tuning was a better strategy than only Transfer Learning, unlike D1 with 702 images and D3 with 1358 images.

### 4.2. Guided Grad-CAM and Occlusion Analysis Insights AD and PSP Classification

CNNs have been poorly considered in the histopathological study of neurodegenerative diseases and to a lesser extent focused on the training of algorithms at the level of fibrillar lesions such as NFTs. Our study points out that Transfer Learning demonstrates strong predictive performance. Therefore, the models developed with ResNet-IFT can implement a criterion of interpretability aided by Guided Grad-CAM and Occlusion Analysis to study and identify structural differences with IPMB images of AD and PSP.

The use of Guided Grad-CAM and Occlusion Analysis showed that the presence of the main tangle in the images, except in the hippocampal region with AD immunoreactive to AT8 antibody, is relevant for classifying it as AD or PSP. The main difference for the classification of these tauopathies in the hippocampal region is that the most relevant structural features of PSP are located in the center of the quadrant despite the biomarker and for AD they are located in the center and periphery of the image.

In the entorhinal cortex, the criteria focus mainly on the most prominent NFT for classification of AD or PSP despite its location in the image. These results represent a novel way to explore and understand the phenomenon of neurodegenerative diseases from immunostaining with specific biomarkers, since they show relevant information that is not salient in the original images, in contrast to the status quo in related research that focuses on the most complex NFT structures evidenced by immunoreactivity [62,63,64].

Hence, we provide additional criteria for the identification of AD that shows that there is relevant information on the periphery of the image. In addition, we note that whenever we have images of both the entorhinal cortex and the hippocampus for AD classification, peripheral structures in the image are relevant.

Tang et al. [29] explain that a limitation with the Guided Grad-CAM algorithm is that it may highlight features of the image that indicate that something is not present for the classification. However, here we are not classifying objects within the image, we are classifying NDs using the most relevant features of an image. If the highlighted portion of the image means the absence of something in comparison to the other class, it still means that it is an important area to look at while researching the pathogenesis of NDs. Moreover, in order to make the research more robust, as a future work we can classify neuron areas or lession traits for each ND to test the reliability of the ResNet-IFT model.

The heat zones assigned by Occlusion Analysis indicate a stronger association with both pS396 antibody immunoreactive areas and TR-positive staining than with AT8 antibody immunoreactivity (Figure 11a–c) in the NFTs in the process of maturing to fibrillar forms (by their circular morphology) located in the hippocampal area of AD patients. This result is in agreement with other studies regarding the aggregation process of the Tau polypeptide, which indicates late phosphorylation at amino acid serine 396 as one of the most advanced events for its polymerization and maturation into insoluble fibrillar forms that have affinity for the Thiazine red molecule [18,65,66].

It is likely that earlier events in pathological processing toward the amino-terminal end of the Tau polypeptide, such as phosphorylation at amino acid serine 202 and threonine 305, show less association than later events toward the carboxyl-terminal end [67]. According to the hot spots assigned by the Occlusion Analysis, even the algorithm discovers other localized areas outside the NFTs, evidenced by immunoreactivity with the AT8 antibody. We consider these data very relevant because they show that other areas independent of immunoreactivity with antibodies directed towards the amino-terminal end may be compromised or associated with the early stages of pathological processing of the Tau protein in the hippocampus of AD patients.

Importantly, we did not observe the same behavior in hippocampal NFTs in PSP patients (Figure 11d–f). For both D1 and D3 training, heat zones are associated with the area that is immunoreactive with AT8 biomarkers, pS396 and Thiazine red dye staining. These data suggest differential processing in Tau polypeptide pathogenesis between PSP and AD tauopathies in their early stages if we analyze hippocampal NFT populations.

In summary, this evidence underlines the importance of the analysis by the occlusion algorithm for our further studies using early and late biomarkers in the pathological processing of Tau protein directed towards its amino-terminal and carboxyl-terminal end, respectively, which can be validated among different neurodegenerative diseases that have the common factor of pathological PTMs of the Tau polypeptide [68].

Regarding the Occlusion Analysis in the NFTs of the entorhinal cortex of patients with AD and PSP (Figure 12), using the same Tau biomarker scheme between both tauopathies, the heat map only points out areas within the regions that are immunoreactive with AT8 antibodies, pS396 and Thiazine red molecule staining with D2.

However, the results obtained with the algorithm trained with D2 are more associated with mature NFTs than those trained with D3, which could suggest that there are molecular differences between initiating and advanced events for these fibrillar structures. Another interesting result with Occlusion Analysis with D3 denotes areas outside the immunoreactive zones in AD (Figure 11a–c) and not in PSP (Figure 11d–f) in the analysis with D3, which confirms possible molecular differences in Tau processing between both proteinopathies in populations of NFTs in the entorhinal cortex.

Still, the common areas found with Occlusion Analysis and Guided Grad-CAM are considered decisive for the classification of PSP and AD. This means that experts could focus their attention more specially on some areas of the image which could save research time. Importantly, clinical profile and histopathological analysis were key factors in selecting patients from our study; however, we have as a perspective to include an analysis of ML/DL algorithms trained with specific markers for PSP and EA, different from Tau biomarkers, in order to compare other variables in prediction methods.

## 5. Conclusions

In this work, we obtained a CNN pipeline using ResNet-IFT architecture and XAI algorithms Guided Grad-CAM and Occlusion Analysis where the classification of AD and PSP in IPMB images achieved an accuracy of 98.41%, on average, using Transfer Learning. We conclude that in cases in which we want to use Transfer Learning and fine-tuning with a ResNet-50-based model, we may need to initialize weights from a similar domain. However, using Transfer Learning with a ResNet-50-based model pre-trained on the ImageNet dataset results in very effective models for classification of AD and PSP in IPMB images.

Our study shows that there may be different structural patterns in the immunoreactivity of the Tau protein in NFTs present in the brains of patients with PSP and AD, as identified with our models. Moreover, our work suggests that DL classification algorithms based on ResNet 50 can support the structural analysis of Tau polypeptide aggregation, which has been studied primarily with histopathological assays for decades. Based on antibody training that has been documented in the advanced stages of pathological Tau processing, the analysis of Guided Grad-CAM and Occlusion Analysis proposes immunoreactive areas of the neurofibrillary tangle and other areas of the quadrant that may be important for studying the aggregation behavior of this protein in AD and PSP. This methodology proposes that to study these diseases, criteria of shared spatial invariability can also be considered between the images that support the CNN models for the IPMB images. While structural patterns are identified in this study, further research is needed to identify the exact nature of this difference.

This first study allows us to suggest that classifier models based on ResNet-50 architectures are valuable for the classification of AD and PSP IPMB images. Finally, these tools will help us to structure the following analyses using close-ups of images to classify AD and PSP using fluorescence images with antibodies against other PTMs that are associated with the formation of Tau filaments, such as conformational changes or endogenous proteolysis. Likewise, we propose to build a multiclass classifier where we carry out the study of structural characteristics comparing PTMs against non-fibrillar Tau controls. Moreover, we are considering whether, in our future work, to combine classification with object detection in order to classify neurodegenerative diseases and also to detect pre-defined features of interest.

## Figures and Tables

**Table 1:** Summary of datasets.

Dataset Label	Brain Regions Included	Classes	Number of Images per Class	Total Images
D1	Hippocampus	Alzheimer	346	656
		PSP	310	
D2	Entorhinal cortex	Alzheimer	393	702
		PSP	309	
D3	Hippocampus and Entorhinal cortex	Alzheimer	739	1358
		PSP	619	

**Table 2:** Summary of CNN Architectures tested.

Architecture Label	Structure	Pretrained	Transfer/Fine Tuning
ResNet-IFT	48-convolution layers	Yes, ImageNet	Transfer
	1 Global Average Pooling		
	1 Dense Layer		
ResNet-IFTF	49-convolution layers	Yes, ImageNet	Transfer and Fine Tuning
	1 Global Average Pooling		
	1 Dense Layer		
Simple CNN	3 blocks of convolution layer and max pooling	No	No
	2 Dense Layer		
Sequential CNN	3 Dense layers	No	No

**Table 3:** Performance evaluation of CNN architectures.

Architecture Label	Dataset	Accuracy
ResNet-IFT	D1	97.55 ± 2.63
	D2	99.29 ± 1.00
	D3	98.38 ± 1.58
ResNet-IFTF	D1	98.18 ± 2.12
	D2	98.57 ± 1.17
	D3	97.20 ± 2.83
Simple CNN	D1	52.00 ± 6.71
	D2	53.42 ± 6.14
	D3	51.11 ± 4.35
Sequential CNN	D1	52.60 ± 1.76
	D2	56.41 ± 1.07
	D3	52.36 ± 3.17

## Data Availability

The data that support the findings of this study are available from National Biobank of Dementias of the National Autonomous University of Mexico (UNAM) but restrictions apply to the availability of these data, which were used under license for the current study and so are not publicly available. Data are however available from the authors upon reasonable request and with permission of National Biobank of Dementias of the National Autonomous University of Mexico (UNAM).

## Abbreviations

The following abbreviations are used in this manuscript:

NDs	Neurodegenerative diseases
AD	Alzheimer’s Disease
PSP	Progressive Supranuclear Palsy
CSF	Cerebrospinal Fluid
PTMs	Post-translational Modifications
CNN	Convolutional Neural Networks
MRI	Magnetic Resonance Imaging
CT	Computerized Tomography
PET	Positron Emission Tomography
IPMB	Immunofluorescence Post-Mortem Brain
XAI	Explainable Artificial Intelligence
UNAM	National Autonomous University of Mexico
